# Maternal-to-fetal allopurinol transfer and xanthine oxidase suppression in the late gestation pregnant rat

**DOI:** 10.1002/phy2.156

**Published:** 2013-11-13

**Authors:** Andrew D Kane, Emily J Camm, Hans G Richter, Ciara Lusby, Deodata Tijsseling, Joepe J Kaandorp, Jan B Derks, Susan E Ozanne, Dino A Giussani

**Affiliations:** 1 Department of Physiology, Development and Neuroscience, University of CambridgeCambridge, United Kingdom; 2 University of Cambridge Metabolic Research Laboratories and MRC Metabolic Diseases Unit, Institute of Metabolic Science, Addenbrooke's HospitalCambridge, United Kingdom; 3 Perinatal Center, University Medical CenterUtrecht, The Netherlands

**Keywords:** Allopurinol, fetus, oxypurinol, xanthine oxidase

## Abstract

Fetal brain hypoxic injury remains a concern in high-risk delivery. There is significant clinical interest in agents that may diminish neuronal damage during birth asphyxia, such as in allopurinol, an inhibitor of the prooxidant enzyme xanthine oxidase. Here, we established in a rodent model the capacity of allopurinol to be taken up by the mother, cross the placenta, rise to therapeutic levels, and suppress xanthine oxidase activity in the fetus. On day 20 of pregnancy, Wistar dams were given 30 or 100 mg kg^−1^ allopurinol orally. Maternal and fetal plasma allopurinol and oxypurinol concentrations were measured, and xanthine oxidase activity in the placenta and maternal and fetal tissues determined. There were significant strong positive correlations between maternal and fetal plasma allopurinol (*r* = 0.97, *P* < 0.05) and oxypurinol (*r* = 0.88, *P* < 0.05) levels. Under baseline conditions, maternal heart (2.18 ± 0.62 mU mg^−1^), maternal liver (0.29 ± 0.08 mU mg^−1^), placenta (1.36 ± 0.42 mU mg^−1^), fetal heart (1.64 ± 0.59 mU mg^−1^), and fetal liver (0.14 ± 0.08 mU mg^−1^) samples all showed significant xanthine oxidase activity. This activity was suppressed in all tissues 2 h after allopurinol administration and remained suppressed 24 h later (*P* < 0.05), despite allopurinol and oxypurinol levels returning toward baseline. The data establish a mammalian model of xanthine oxidase inhibition in the mother, placenta, and fetus, allowing investigation of the role of xanthine oxidase–derived reactive oxygen species in the maternal, placental, and fetal physiology during healthy and complicated pregnancy.

## Introduction

Despite advances in obstetric practice, acute intrapartum fetal hypoxia remains one of the most common forms of fetal stress, with substantial morbidity and mortality (Low 2004). One possible strategy to combat the detrimental effects of fetal hypoxia would be to decrease the associated excessive generation of reactive oxygen species (ROS) from stimulated prooxidant mechanisms within the cell, such as the xanthine oxidase pathway (Berry and Hare 2004). Indeed, maternal treatment with the xanthine oxidase inhibitor allopurinol is being considered in human pregnancy complicated by intrapartum hypoxia in order to protect the infant from excessive generation of ROS (Peeters-Scholte et al. 2003; Benders et al. 2006; Chaudhari and McGuire 2008; Torrance et al. 2009; Kaandorp et al. 2010, 2011b, 2012). This clinical interest in antenatal maternal administration of allopurinol follows previous studies which reported that allopurinol treatment in the asphyxiated neonate improved neonatal outcome (Van Bel et al. 1998), but if the time interval between hypoxia and treatment had been too long, or when fetal hypoxia had been too severe, no reduction in serious morbidity or mortality was observed (Benders et al. 2006). Therefore, there has been growing clinical and scientific interest in establishing whether perinatal outcome may be improved in complicated labor if the window of treatment with allopurinol could be initiated before birth, for instance, via maternal treatment to cover the actual period of fetal hypoxia and reperfusion (Derks et al. 2010; Kaandorp et al. 2010).

Whereas there have been studies in large animals including the pregnant ewe (Masaoka et al. 2005; Derks et al. 2010) and sow (van Dijk et al. 2008), no small animal model of allopurinol administration to the mother during pregnancy has been established. Such a model is indispensable in species with a comparatively shorter life span to allow follow up of the effects of xanthine oxidase inhibition during pregnancy on the physiology of the offspring in later life. This is essential when many of the conditions associated with a suboptimal fetal environment such as cardiovascular disease and type 2 diabetes only emerge in later life.

Therefore, the aims of this study were to investigate in rodent pregnancy: (1) whether xanthine oxidase is active in the placenta, maternal, and fetal tissues in late gestation; (2) if maternal oral administration of allopurinol could increase maternal plasma levels of allopurinol and its active metabolite oxypurinol; (3) if maternal allopurinol and oxypurinol could cross the placenta, yielding therapeutic levels in the fetal circulation; and (4) whether elevations in maternal and fetal plasma levels of allopurinol and oxypurinol resulted in suppression of xanthine oxidase activity in the placenta, maternal, and fetal tissues. These aims were determined using a clinically relevant dose of allopurinol (30 mg kg^−1^), comparable to the dose given in previous human studies (Van Bel et al. 1998; Benders et al. 2006), and a larger dose (100 mg kg^−1^) to investigate any pharmacological benefit in terms of either greater fetal levels of allopurinol and/or longer inhibition of xanthine oxidase activity.

## Materials and Methods

All procedures involving animals were carried out under the Animals (Scientific Procedures) Act 1986 and approved by the Local Ethics Review Committee of the University of Cambridge, U.K. Thirty-five time-mated pregnant Wistar rats (Charles River Limited, Margate, U.K.) were delivered to the University of Cambridge between 10 and 14 days of gestation and were individually housed under standard conditions (21 ± 1°C, 55% humidity, and 12 h/12 h light/dark cycle) with free access to food and water.

On day 20 of pregnancy all animals were randomized to either control or allopurinol treatment. Allopurinol (30 mg kg^−1^ or 100 mg kg^−1^, Sigma, U.K., suspended in 3 mL Hartley's Strawberry Jelly) was placed in a clear glass bowl inside the cage at 8 am. Animals had been given an untreated jelly dose the day before to condition them to eat it. Following allopurinol treatment, animals underwent euthanasia (CO_2_ followed by cervical dislocation) at 2, 6, or 24 h following administration (*n* = 5 all time points and doses). A further five animals underwent euthanasia at 8 am on day 20 of pregnancy to act as controls. Following euthanasia, a maternal blood sample was taken by cardiac puncture for the measurement of maternal circulating allopurinol and oxypurinol levels. Fetal rats were then exposed by laparotomy. The fetuses were subjected to cervical transection, and blood was collected and pooled from all fetuses. Maternal and fetal blood samples were anticoagulated with ethylenediaminetetraacetic acid (EDTA), centrifuged (3000 *g* for 5 min), and the plasma was frozen in liquid nitrogen and subsequently stored at −80°C until analysis for allopurinol and oxypurinol. Samples of placenta, maternal heart, maternal liver, and fetal liver of ca. 100 mg were taken, weighed, and homogenized in buffer (1 mL of 100 mmol L^−1^ TRIS-HCl, pH 7.5 with 10 *μ*L protease inhibitor cocktail, SIGMA, P8340). Fetal heart samples were ca. 20–25 mg. All further analysis was adjusted for the weight of tissue taken. Fetal tissue samples and associated placentas were taken only from male pups to reduce any differences attributable to sex. The sex of each fetal pup was determined by their anogenital distance as previously described (Camm et al. 2010). Following centrifugation of the tissue homogenate (30 min, 10,000 *g* at 4°C), the supernatant was decanted and frozen in aliquots at −80°C until further analysis.

### Plasma allopurinol and oxypurinol measurements

Allopurinol and oxypurinol plasma concentrations were determined by using reversed-phase, high-performance liquid chromatography with ultraviolet detection at 254 nm for the quantification of allopurinol and oxypurinol in plasma (van Kesteren et al. 2006). The method was linear between 0.5 and 25 *μ*g mL^−1^ with a lower limit of detection of 0.2 *μ*g mL^−1^ for both compounds.

### Xanthine oxidase assay

Fetal plasma xanthine oxidase activity levels were measured using a commercially available assay kit (A22182; Invitrogen, Paisley, U.K.). The assay works on the principle that xanthine oxidase produces superoxide anions. In vitro, superoxide spontaneously degrades to hydrogen peroxide (H_2_O_2_). H_2_O_2_, in the presence of horseradish peroxidase (HRP), reacts stoichiometrically with Amplex Red reagent to generate the red-fluorescent oxidation product, resorufin. In brief, 50 *μ*L of tissue homogenate or xanthine oxidase standard solution was added to 50 *μ*L of 100 *μ*mol/L Amplex Red reagent solution which also contained 0.4 U mL^−1^ HRP and 200 *μ*mol/L hypoxanthine. The resulting solutions were mixed and then incubated for 30 min at 37°C. The presence of resorufin was detected by fluorescence using excitation in the range 530–560 nm and emission detection at 590 nm. Activity was then expressed per mg of wet tissue.

### Data and statistical analysis

All values are expressed as mean ± SEM. The correlation between fetal and maternal allopurinol and oxypurinol levels was assessed using the Pearson product moment correlation (Sigma-Stat 3.5; Chicago, IL). Allopurinol, oxypurinol and xanthine oxidase activity levels were compared using one-way analysis of variance (ANOVA) comparing with reference to time 0 h. Where significant differences were found, the Student–Newman–Keuls post hoc test was applied (Sigma-Stat 3.5; Chicago, IL). Statistical significance was accepted when *P* < 0.05.

## Results

### Allopurinol and oxypurinol levels in maternal and fetal plasma

**Figure 1:**
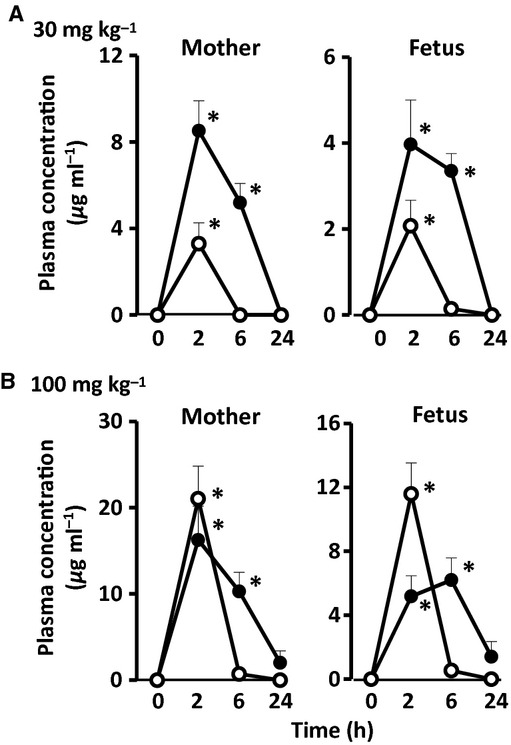
Maternal and fetal plasma allopurinol and oxypurinol measurements. Values are mean ± SEM for allopurinol (○) and oxypurinol (•) concentrations in maternal and fetal plasma (*n* = 5 per time point and group) at 0, 2, 6, and 24 h postadministration of (A) 30 mg kg^−1^, and (B) 100 mg kg^−1^ of allopurinol to the mother at time 0 h. One-way ANOVA with post hoc Student–Newman–Keuls where appropriate. Significant differences (*P* < 0.05): *, versus 0 h control.

Administration of 30 mg kg^−1^ and 100 mg kg^−1^ allopurinol to the pregnant rat led to increases in both maternal and fetal allopurinol and oxypurinol by 2 h (Fig. 1). Following the administration of allopurinol at both doses of 30 mg kg^−1^ and 100 mg kg^−1^, allopurinol levels returned to baseline at 6 h following dosing; however, oxypurinol levels remained significantly elevated in both mother and fetus. Although maternal and fetal plasma oxypurinol levels were detectable 24 h following dosing, they were not significantly different from baseline.

**Figure 2:**
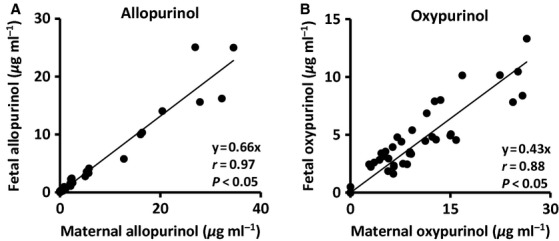
Maternal to fetal allopurinol and oxypurinol correlations. Values are paired maternal and fetal (A) allopurinol and (B) oxypurinol concentrations at all time points and both 30 mg kg^−1^ and 100 mg kg^−1^ allopurinol dosing protocols. *N* = 35 paired samples per analysis. Both relationships show significant positive correlation (*P* < 0.05, Pearson correlation).

When all paired maternal and fetal plasma concentrations of allopurinol and oxypurinol were related to determine maternal-to-fetal transfer, a significant positive correlation was obtained for both compounds (*P* < 0.05; Fig. 2). The correlation coefficient (*r*) for the relationship between maternal and fetal allopurinol was 0.97, and between maternal and fetal oxypurinol was 0.88, indicating a high degree of correlation (Fig. 2).

### Plasma xanthine oxidase levels

**Figure 3:**
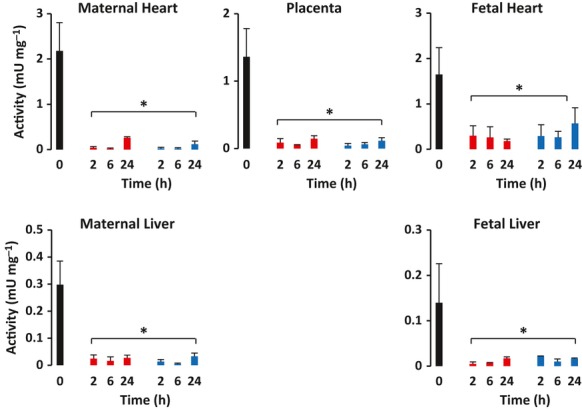
Maternal, fetal, and placental xanthine oxidase activity. Values are mean ± SEM for tissue xanthine oxidase activity in samples of maternal heart and liver, planceta, fetal heart, and liver maternal and fetal organ activities of xanthine oxidase assayed in vitro at 0, 2, 6, and 24 h following oral administration of allopurinol to the pregnant dam on day 20 of pregnancy (▪, 0 h control; 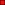, 30 mg kg^−1^; 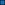, 100 mg kg^−1^; *n* = 5 per time point). One-way ANOVA with post hoc Student–Newman–Keuls where appropriate. Significant differences (*P* < 0.05): *, versus 0 h control.

Xanthine oxidase activity was detectable in maternal heart, maternal liver, placenta, fetal heart, and fetal liver (Fig. 3). Maternal and fetal cardiac tissue displayed the greatest activity (2.17 ± 0.6 mU mg^−1^ and 1.65 ± 0.59 mU mg^−1^, respectively), followed by placenta (1.36 ± 0.41 mU mg^−1^), and then maternal liver and fetal liver (0.29 ± 0.09 mU mg^−1^ and 0.14 ± 0.08 mU mg^−1^). Treatment with allopurinol at 30 mg kg^−1^ and 100 mg kg^−1^ significantly depressed activity in all tissues by 2 h and this suppression was sustained 6 and 24 h following treatment.

## Discussion

Despite intense basic science research and clinical trials investigating the potential neuroprotective effects of the xanthine oxidase inhibitor allopurinol on the fetal brain during and following acute fetal hypoxia (Boda et al. 1984; Van Bel et al. 1998; Benders et al. 2006; Derks et al. 2006, 2010; van Dijk et al. 2008; Kaandorp et al. 2010, 2011a,b), no animal model exists that offers the possibility of maternal administration of allopurinol and long-term follow up of the treated offspring. Here, we show that allopurinol administered orally to the pregnant rat during late pregnancy leads to therapeutic rises in circulating allopurinol and oxypurinol in the mother and fetus. The increased levels achieved were functionally relevant as demonstrated by significantly reduced, xanthine oxidase activity levels in the placenta, and maternal and fetal tissues following 24 h of maternal allopurinol administration. The level of suppression of xanthine oxidase activity was similar with the low and high doses of maternal allopurinol administration.

Currently, human clinical studies based in the Netherlands are assessing whether antenatal allopurinol administration during intrapartum fetal hypoxia can reduce overall morbidity and mortality in the newborn (Kaandorp et al. 2010, 2012, 2013). Recent follow-up data from those trials suggest that there may be a reduction in the relative risk of severe disability between 4 and 8 years of age in children who were moderately asphyxiated intrapartum but received postnatal allopurinol treatment starting within 4 h after birth (Kaandorp et al. 2012). Importantly, postnatal allopurinol was not associated with any negative side effects in the children who were followed up (Kaandorp et al. 2012). The rationale behind moving clinical therapy from postnatal to antenatal allopurinol administration is that by shortening the period between the beginning of ROS production and the antioxidant cover, the overall tissue injury, and thereby clinical disability, may be reduced. In support of this hypothesis, we have recently reported that maternal antenatal allopurinol administration can reduce hippocampal brain damage in an ovine model of repeated birth asphyxia (Kaandorp et al. 2013).

Previous reports have confirmed that maternal treatment with allopurinol crosses the ovine (Masaoka et al. 2005; Derks et al. 2010), porcine (van Dijk et al. 2008), and human (Boda et al. 1999; Torrance et al. 2009) placenta to increase fetal plasma concentrations of allopurinol and oxypurinol. In sheep, a dose of 20 mg kg^−1^ i.v. to the maternal ewe led to peak maternal allopurinol and oxypurinol concentrations of 47 and 17 *μ*g mL^−1^, respectively, and allopurinol concentrations of approximately 5 *μ*g mL^−1^ in the fetus (Derks et al. 2010). In a pregnant sow and piglet model, 15 mg kg^−1^ allopurinol given intravenously to the mother led to fetal peak concentrations of 5 *μ*g mg^−1^ (van Dijk et al. 2008). In this study, the plasma levels of allopurinol and oxypurinol reached 3.3 *μ*g mg^−1^ and 8.5 *μ*g mg^−1^, respectively, in maternal plasma and 2.1 *μ*g mg^−1^ and 4.0 *μ*g mg^−1^, respectively, in fetal plasma for the 30 mg kg^−1^ oral dose, values that are slightly lower than those measured in the sheep and pig for doses of similar magnitude. This may reflect species differences in the pharmacokinetics of allopurinol (Goodman et al. 2005). For example, there may be reduced bioavailability of allopurinol and oxypurinol following oral administration relative to other routes of administration and/or differences in drug transfer across the placenta. It is also possible that basal xanthine oxidase activity in the rodent dam and its fetus may be different, altering the rate of conversion of allopurinol into oxypurinol. Finally, differences in the clearance of allopurinol and oxypurinol from the circulation may also exist. Following treatment with 100 mg kg^−1^ of allopurinol to the mother, fetal values of allopurinol and oxypurinol were 11.6 *μ*g mg^−1^ and 5.2 *μ*g mg^−1^ at 2 h, respectively. It might have been expected that these values would be approximately three times greater than those recorded following 30 mg kg^−1^. However, given that xanthine oxidase catalyzes the conversion of allopurinol to oxypurinol, and that this reaction is prevented by oxypurinol as the active metabolite, the production of oxypurinol may prevent conversion of allopurinol into oxypurinol, leading to a relatively high allopurinol concentration and therefore decreasing the rate of further generation of oxypurinol (Parks and Granger 1986; Berry and Hare 2004). There was also a high correlation between paired maternal and fetal concentrations of allopurinol and oxypurinol, independent of time point of dose, indicating that placental transfer is reliable and consistent. However, fetal levels of allopurinol and oxypurinol were lower than maternal, reflected by the coefficient of the straight line relationship being <1.

Effective inhibition of xanthine oxidase has been previously reported in the adult rat over the range of allopurinol doses from 2 to 50 mg kg^−1^ (Klein et al. 1996). In the sheep fetus, concentrations of allopurinol of 2.25 *μ*g mL^−1^ achieved a lowering of ROS production (Masaoka et al. 2005), and 5 *μ*g mL^−1^ allopurinol increased umbilical blood flow following umbilical cord occlusion (Derks et al. 2010). In ovine pregnancy, larger doses of allopurinol led to suppression of *α*_1_-mediated vasoconstriction in the fetal femoral vascular bed via decreasing ROS-mediated NO depletion in the circulation (Herrera et al. 2012). In this study, allopurinol concentrations as low as 2.1 *μ*g mL^−1^ were sufficient to inhibit xanthine oxidase activity in the fetal rat, with higher doses not offering greater inhibition. Despite the decrease in allopurinol and oxypurinol levels over the 24 h period examined in this experiment, xanthine oxidase activity was still suppressed in all tissues investigated. Therefore, doses of allopurinol as low as 30 mg kg^−1^ administered to the mother are effective to promote the long-term suppression of xanthine oxidase activity in placenta and the fetal organs.

In summary, this study in rodent pregnancy has established a human clinically relevant model of maternal allopurinol administration that leads to therapeutic concentrations of allopurinol and oxypurinol in the fetal circulation. A single dose of 30 mg kg^−1^ allopurinol is sufficient to suppress xanthine oxidase activity in the mother, placenta, and fetus for at least 24 h. The model is indispensable to investigate the long-term consequences of maternal treatment with allopurinol on the physiology of the offspring, and to establish the lasting safety of xanthine oxidase inhibition in human high-risk pregnancy in current clinical obstetric practice.
